# Successful Management of a Keratoconus Patient With Rarely Used Large-Diameter Corneal RGP Contact Lenses: A Case Report

**DOI:** 10.7759/cureus.77801

**Published:** 2025-01-21

**Authors:** Ahmed Almaweri

**Affiliations:** 1 Department of Optometry, Noor Alyemen Eye and E.N.T. Consulting Center, Sana'a, YEM

**Keywords:** case report, contact lenses, corneal, diameter, keratoconus

## Abstract

Keratoconus is a degenerative corneal disease that is usually bilateral and asymmetric. In keratoconus, the cornea progressively thins and steepens, leading to vision impairment. This unique case report highlights the management of a keratoconus patient with infrequently used large-diameter corneal RGP contact lenses. This method of fitting provides remarkable stability and patient comfort. Here, I describe a case of a 39-year-old man with a known history of keratoconus. He was referred to our contact lens clinic for management with RGP lenses. Throughout the eye exam, the patient was deemed eligible to wear RGP contact lenses. Uncorrected visual acuity was 20/400 in the right eye and 20/400 in the left eye. Visual acuity improved to 20/40 in the right eye with a correction of -6.00/-3.00 x 90 and to 20/30 in the left eye with a correction of -6.00/-2.00 x 95. I fitted this patient with large-diameter (10.30 mm) corneal RGP lenses in both eyes, and the fitting was optimal, resulting in a significant improvement in visual acuity to 20/20 in each eye. This case study aims to document the success of large-diameter corneal RGP contact lenses in the management of keratoconus.

## Introduction

Keratoconus is a degenerative corneal disease that is usually bilateral and asymmetric. In keratoconus, the cornea progressively thins and steepens, deviating from its normal shape and leading to vision impairment [1]. Keratoconus displays no ethnic or gender preference. It is primarily an ocular condition but can also be associated with other eye and systemic diseases [1]. Although the initiation of keratoconus is most common during late childhood and puberty, it is more frequently diagnosed in early adulthood. The condition progresses for one to two decades, generally stabilizing by the fourth decade of life [2]. A study performed in Saudi Arabia revealed a high rate of keratoconus in young individuals aged six to twenty-one years. Approximately 4.8% of patients visiting specialized hospitals for non-ocular reasons were unexpectedly diagnosed with this condition [2].

The management of keratoconus varies depending on its severity. Mild cases often respond to eyeglasses, while moderate to advanced cases may require RGP contact lenses. In severe conditions that can't be effectively treated with specialized contact lenses, such as scleral lenses, corneal transplantation might be needed [3].

Rigid gas permeable (RGP) contact lenses, first introduced by Adolf Fick in 1888, continue to be the supreme choice for correcting vision in keratoconus patients to this day. They reshape the irregular corneal surface, optimizing visual acuity and minimizing aberrations. In comparison with glasses, RGP lenses offer superior visual quality and quantity [4].

Corneal RGP contact lenses are made from rigid gas-permeable materials. They are lenses that rest entirely on the cornea, with a total diameter that does not exceed the limbus. Corneal RGP contact lenses are classified by their total diameter into three main classes: small, medium, and large. Small lenses have a diameter between 8.50 mm and 9.30 mm, medium lenses fall within the range of 9.40 mm to 9.90 mm, and large lenses have a diameter of 10.00 mm and above.

The purpose of this case study is to document the success of less commonly used large-diameter corneal RGP contact lenses for keratoconus management. This case report provides valuable insights for contact lens professionals considering large-diameter RGP lenses as a management option for keratoconus patients.

## Case presentation

A 39-year-old man was referred to our contact lens clinic from a nearby hospital. The patient presented with a diagnosis of keratoconus. He had a history of corneal collagen cross-linking (CXL) performed two years ago. Additionally, there was no family history of keratoconus or personal history of vernal keratoconjunctivitis (VKC). The patient has a history of well-controlled type 2 diabetes mellitus for ten years, with an otherwise unremarkable medical history.

Prior to the contact lens fitting, the patient underwent a routine eye examination at our center. These examinations included a detailed assessment of visual acuity, both with and without correction, as well as objective and subjective refraction. A comprehensive slit-lamp evaluation of the anterior eye segment was performed, including the lids, conjunctiva, cornea, iris, lens, and anterior chamber. Fundoscopy was also conducted to evaluate the health of the posterior segment, including the retina, optic nerve, vessels, and macula.

The patient's uncorrected visual acuity was 20/400 in the right eye and 20/400 in the left eye. With the best spectacle correction, visual acuity improved to 20/40 in the right eye with a prescription of -6.00/-3.00 x 90 and 20/30 in the left eye with a prescription of -6.00/-2.00 x 95. The visual acuity with a pinhole was 20/50 in both eyes.

Both eyes showed a palpebral aperture fissure height of 11 mm, a horizontal visible iris diameter of 12 mm, and a vertical visible iris diameter of 11 mm. The pupillary diameter was measured with a ruler. In bright light, the pupil diameter was 3 mm in both eyes. Under dim light, the pupil diameter was 5 mm in both eyes. The lid tension was typical; it was neither tight nor loose. The tear film was within normal limits in terms of quality and quantity.

A slit-lamp examination of the anterior segment of both eyes indicated the classic signs of keratoconus in both eyes; otherwise, all findings were within normal limits. Fundoscopy revealed a healthy posterior segment, with no signs of diabetic retinopathy. A handheld iCare tonometer was used to measure intraocular pressure (IOP) at 10:30 AM. The IOP was 10 mmHg in the right eye and 9 mmHg in the left eye.

The four maps of corneal tomography (Pentacam) provide crucial data that can serve as a primary basis for contact lens fitting, including keratoconus staging and cone morphology.

**Figure 1:**
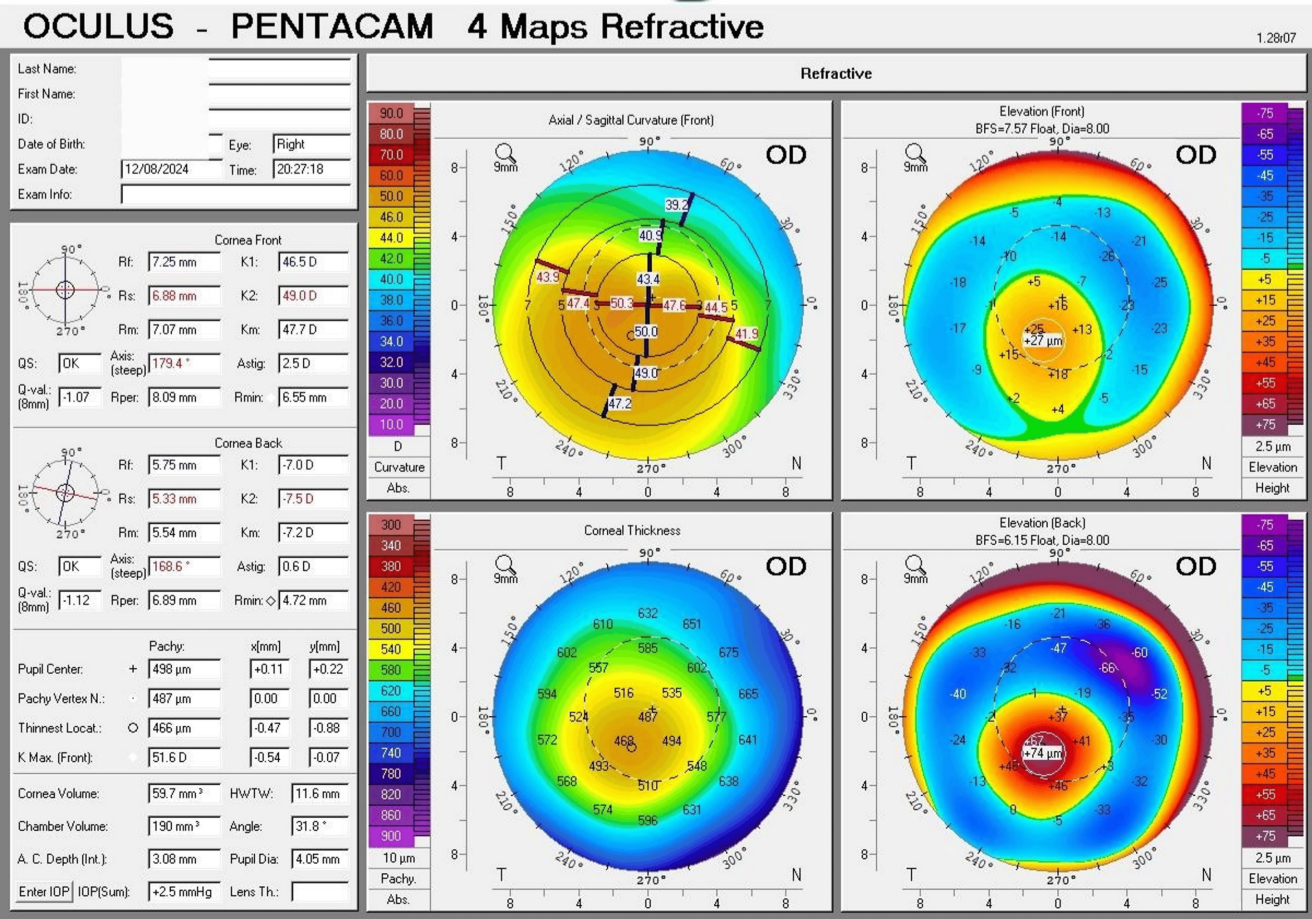
Four maps of Pentacam's corneal tomography for the right eye Four maps of Pentacam's corneal tomography for the right eye display characteristics of moderate (Grade 2) keratoconus. The curvature map reveals an oval cone with inferotemporal translocation of the cone. The thickness map indicates a slightly thin cornea. The anterior and posterior surface elevation maps display abnormal values.

The key data can be presented as follows: the right eye measurements showed a flatter corneal curvature (K1) of 46.5 D (7.25 mm), a steeper corneal curvature (K2) of 49.0 D (6.88 mm), an average corneal curvature (Km) of 47.7 D (7.07 mm), and astigmatism of 2.50 D. The point of the thinnest corneal thickness was 466 µm, while the thickness at the pupil center was 498 µm. The highest point of the anterior surface elevation was 27 µm, and the highest point of the posterior surface elevation was 74 µm (Figure 1).

**Figure 2:**
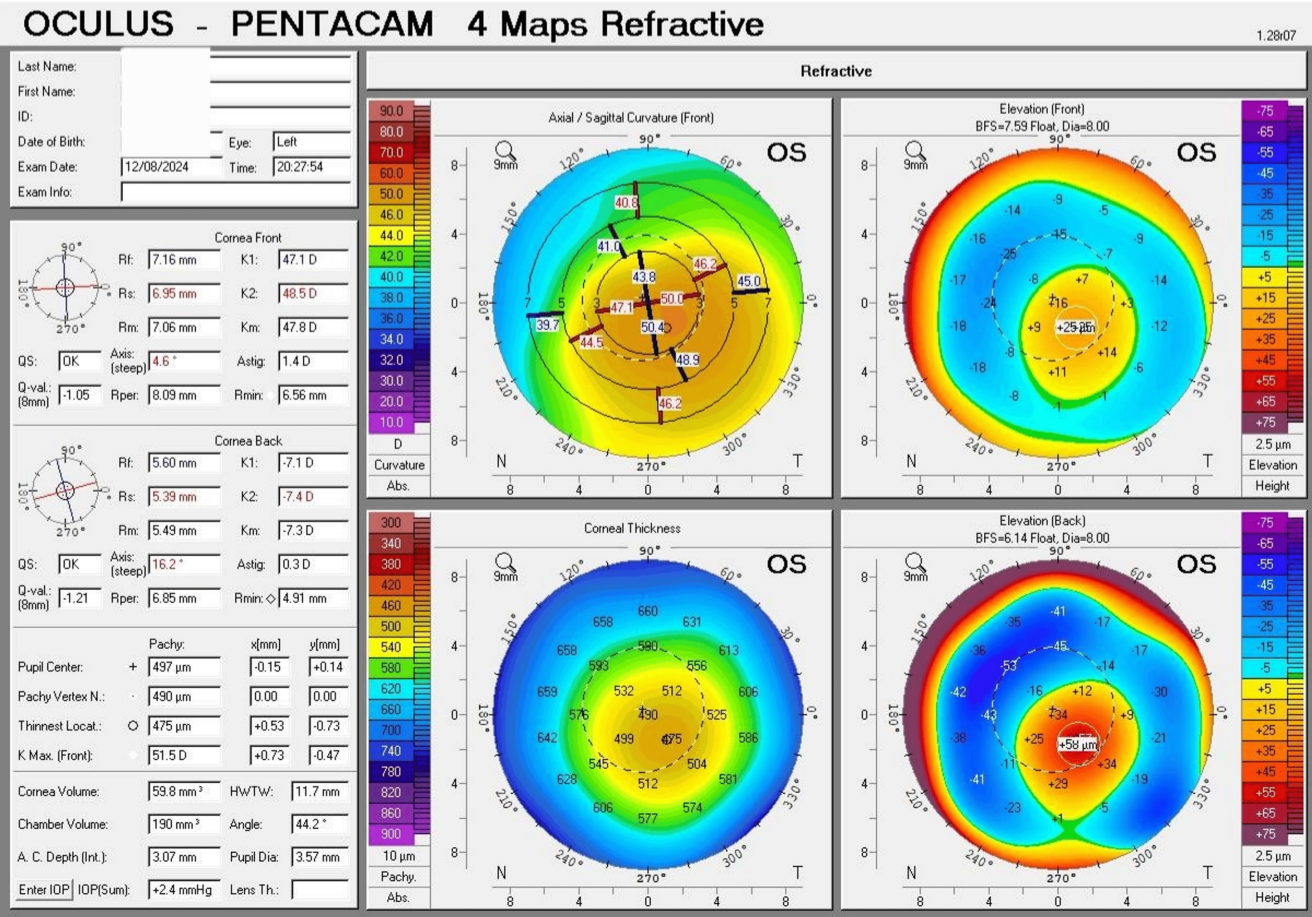
Four maps of Pentacam's corneal tomography for the left eye Four maps of Pentacam's corneal tomography for the left eye exhibit features of moderate (Grade 2) keratoconus. The curvature map shows an oval cone with inferotemporal translocation of the cone. The thickness map indicates a slightly thin cornea. The anterior and posterior surface elevation maps display abnormal values.

The left eye readings exhibited a flatter corneal curvature (K1) of 47.1 D (7.16 mm), a steeper corneal curvature (K2) of 48.5 D (6.95 mm), an average corneal curvature (Km) of 47.8 D (7.06 mm), and astigmatism of 1.40 D. The point of the thinnest corneal thickness was 475 µm, while the thickness at the pupil center was 497 µm. The highest point of the anterior surface elevation was 25 µm, and the highest point of the posterior surface elevation was 58 µm (Figure 2).

Following routine eye examinations conducted at our center, I found the patient eligible to wear contact lenses. There are no contraindications to their use. In order to fit my patient with corneal RGP contact lenses, I employed a diagnostic trial lens approach and an aspherical back-surface design. The trial lens set was provided by TS Lac, an Italian company.

To ensure optimal fitting, topical anesthetic eye drops were administered to each eye. This step was necessary to temporarily minimize reflex tearing and facilitate a more accurate evaluation of the contact lens fit. A comprehensive evaluation of the corneal RGP contact lenses was conducted. I assessed key factors, including lens centration, movement, alignment (geometric fit), over-refraction, and patient comfort. These parameters are essential for optimal lens function and patient satisfaction. A small-diameter corneal RGP contact lens with a diameter of 9.30 mm was selected for the patient, and the base curve of the lens was chosen to closely match the patient's mean corneal curvature (K-mean). These elements provide a good starting point for the lens-fitting steps. I initiated the contact lens fitting process by assessing the centration of a trial lens on the cornea. At first, the small-diameter trial lens, with a diameter of 9.30 mm, exhibited significant decentration, as the lens tended to ride low. To address this issue, I moved forward to a larger 9.80 mm lens, but the low-riding position persisted. To elevate the lens position and achieve optimal centration, I further increased the diameter to 10.30 mm. This final adjustment resulted in excellent centration with ideal lid attachment.

**Figure 3:**
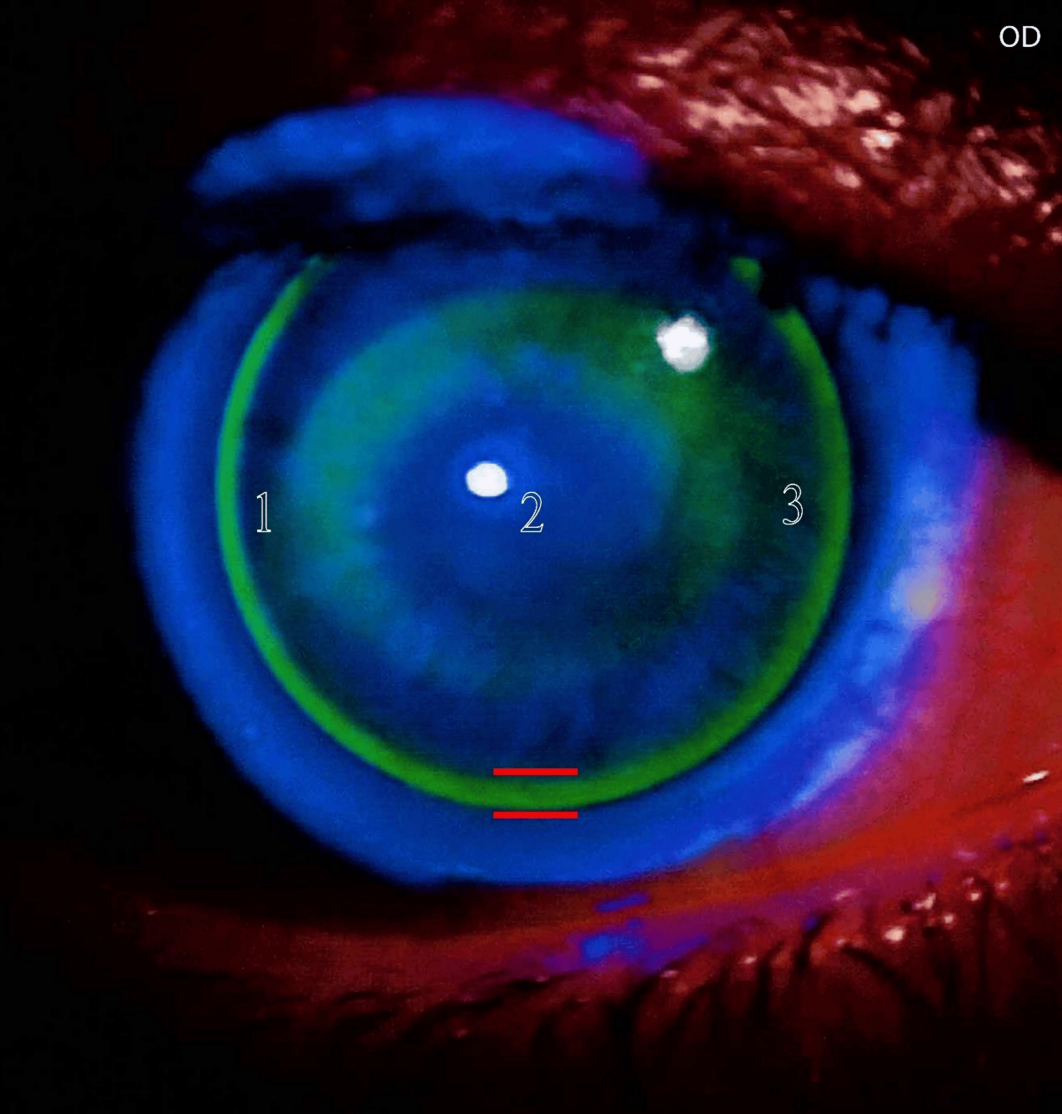
Fluorescein pattern for the right eye The fluorescein pattern for the right eye shows optimal fitting in terms of satisfactory alignment between the RGP lens and the cornea at the center, mid-periphery, and periphery (sufficient edge lift). In this image, edge lift is marked by red lines. This pattern also exhibits excellent lens centration and appropriate lid attachment with a slightly flat fit. This pattern follows the three-point touch fitting philosophy; here, these points are marked by numbers.

**Figure 4:**
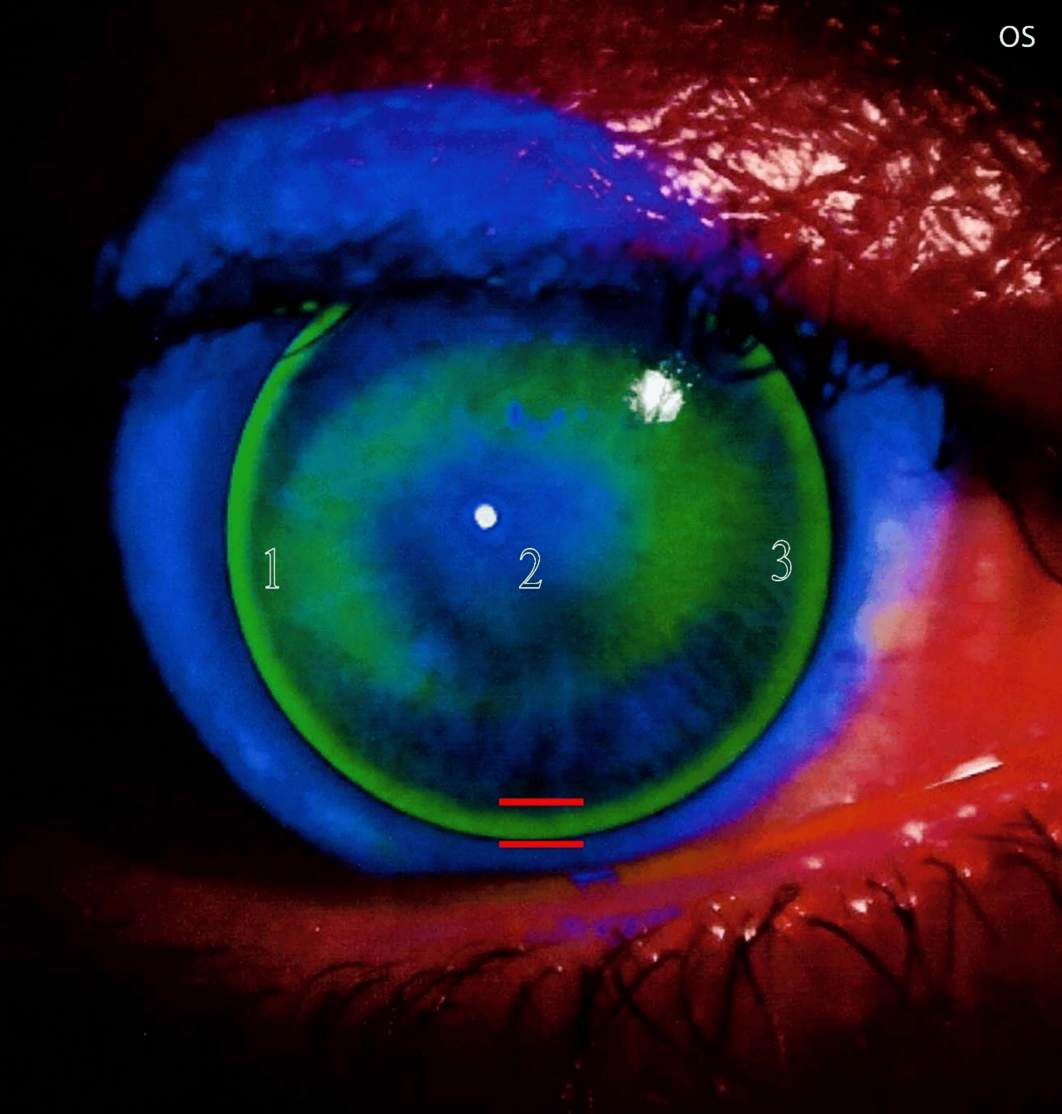
Fluorescein pattern for the left eye The fluorescein pattern for the left eye indicates optimal fitting. The lens is well-centered and properly aligned with the cornea at the center, mid-periphery, and periphery (with adequate edge lift). In this image, edge lift is marked by red lines. The pattern also demonstrates satisfactory lid attachment and a slightly flat fit. This pattern aligns with the three-point touch fitting philosophy; here, these points are marked with numbers.

Following confirmation of optimal lens centration, the evaluation proceeded to the next step of the fitting assessment: lens movement within the eye. Lens movement was evaluated after a blink. The lens movement was found to be optimal, with no signs of restriction or excessive mobility. The fitting assessment continued with the evaluation of lens alignment on the cornea. I began with a base curve equal to the mean corneal curvature (K-mean) as a starting point until I arrived at the best trial lens. In this phase, I assessed the geometric match between the anterior corneal surface and the contact lens back surface. To achieve this evaluation, I used fluorescein strips with a saline solution. Finally, the fluorescein pattern showed optimal fitting with a three-point touch (Figures 3, 4).

In order to obtain the best possible visual outcome, an over-refraction was carefully performed to finalize the appropriate lens back vertex power (BVP). To evaluate the comfort and tolerance of the fitted contact lenses, the patient was asked to wear them for one hour in the waiting room. After the adaptation period, the patient reported a substantial reduction in lens awareness, with no signs of excessive tearing and no pain or ocular irritation.

Upon completion of the contact lens fitting assessment, the optimal lens parameters for the patient were established. The parameters for the right eye include a base curve (BC) of 7.40 mm, a back vertex power (BVP) of -4.00 diopters, a total diameter (TD) of 10.30 mm, and a blue handling tint. For the left eye, the parameters include a base curve (BC) of 7.30 mm, a back vertex power (BVP) of -4.25 diopters, a total diameter (TD) of 10.30 mm, and a gray handling tint.

## Discussion

This case report demonstrates the efficacy of large-diameter corneal RGP contact lenses in managing a keratoconus patient. The use of such large-diameter lenses represents a less frequently employed approach in corneal RGP lens fitting for keratoconus.

While eyeglasses may be adequate in the early stages of keratoconus, contact lenses often become essential as the disease progresses. Soft contact lenses, however, have limitations in keratoconus management. Their inability to correct irregular astigmatism, a hallmark of the disease, and the potential for corneal edema due to the necessary lens thickness render them less suitable. In contrast, rigid gas permeable (RGP) contact lenses provide superior optical quality in keratoconic eyes, making them the preferred option for most patients with this condition [5].

RGP contact lenses provide several advantages. They are oxygen-permeable and more resistant to deposit formation, which supports eye health. In contrast to soft lenses, RGP lenses don't dehydrate the tear film, which assists in comfort. Visually, they provide the best vision with more accurate prescriptions and can be customized to the patient's needs. The rigid nature and design of RGP lenses sustain optical quality because they are not influenced by the shape of the eye. RGP lenses display better durability and a prolonged lifespan due to their high-quality materials [6].

A study conducted by Zadnik et al on the Collaborative Longitudinal Evaluation of Keratoconus (CLEK) revealed that only 16% of keratoconus patients primarily depended on spectacles, while 65% to 75% used contact lenses, mainly rigid gas-permeable contact lenses (RGPCLs). Surgical management was considered for the 10% to 20% of patients who did not respond well to these strategies [7]. A study by Bilgin et al involving 518 keratoconus patients indicated that rigid gas-permeable contact lenses significantly postponed the requirement for surgery in 98.9% of cases [8].

Small-diameter corneal RGP lenses are commonly used in the management of keratoconus. A smaller total diameter leads to a smaller back optic zone diameter (BOZD), which facilitates the fitting of the lens to the conical corneal shape and helps achieve an optimal fit. The back optic zone diameter (BOZD) is a fixed design parameter within a given total diameter (TD), typically measuring 1 to 1.5 mm smaller than the total diameter [9]. Large-diameter corneal RGP contact lenses have major advantages in terms of comfort and stability. Additionally, the larger-diameter RGP lenses exhibit somewhat good movement on the eye. While this movement is less than that of small-diameter lenses, it facilitates tear exchange. This design combines the advantages of both corneoscleral and mini-scleral lenses, as well as smaller corneal RGP lenses [9].

The three-point-touch approach aims to distribute the lens's weight equally across the cornea by lightly touching the apex and two points in the mid-periphery. This minimizes pressure on the sensitive apex, protecting it while ensuring the best vision and optimal tear exchange. While other fitting techniques exist, three-point-touch remains the most commonly used method in contact lens practice [10,11].

Sorbara et al determined that initial comfort was significantly affected by the correlation between lens diameter and cone morphology. Specifically, smaller diameters demonstrated better comfort with centered nipple cones, while larger diameters exhibited higher comfort with decentered oval cones. My study observations support the results of Sorbara et al. In this case study, I fitted a keratoconus patient who had a decentered oval cone with a large diameter (10.30 mm), and the outcome was optimal comfort [12].

The fitting approach employed in this case was lid attachment. This technique is commonly used with large corneal RGP lenses. In the lid attachment fitting modality, the lens edge is positioned beneath the eyelid, enhancing patient comfort by avoiding interaction between the lens edge and the eyelid margin [13].

In this case, I fitted the patient with a slightly flatter fitting to compensate for the increase in sagittal depth as a result of the large diameter. A flatter fitting also provides better visual acuity and minimizes higher-order aberrations [14]. In mild to moderate keratoconus, there is no risk when the fitting is slightly flatter; at this stage, epithelial cells are somewhat tight, making the epithelium resistant to damage. However, in advanced to severe keratoconus, the situation is quite the opposite [15].

I have concluded that the rarity of the approach of using large-diameter corneal RGP lenses is evident from the retrospective analysis of our contact lens clinic records. Of the 100 keratoconic patients fitted with various RGP lenses, including both corneal and scleral designs, the vast majority of cases were managed with small corneal RGP lenses with a diameter of 8.70 mm, and only two keratoconic patients were managed with large corneal RGP lenses with a diameter of 10.30 mm.

Based on my clinical experience in contact lens practice, I believe the reason for the rare use of large-diameter corneal RGP lenses is that patients with keratoconus usually seek RGP contact lens fittings when the condition has advanced, making smaller-diameter lenses a more suitable choice. We commonly use large-diameter corneal RGP lenses for mild to moderate cases, whereas small-diameter lenses are typically recommended for advanced and severe conditions [16].

In order to enhance patient compliance and reduce the likelihood of lens mix-ups, contact lenses were customized with different tints. A light blue tint was selected for the right lens, and a light gray tint was selected for the left lens.

The patient was instructed to have routine follow-up visits. After the fitting, I scheduled close appointments for him. When the fitting was deemed acceptable, follow-up was planned for every six months. During all previous follow-ups, there were no complications or other adverse events. At each visit, I checked vision, fitting, eye health, and the patient's compliance.

In this study, the participant patient reported significant improvement in his ability to perform daily tasks and work-related activities after being fitted with large-diameter corneal RGP lenses. These improvements, coupled with his feedback during follow-up visits, underscore the intervention’s positive impact on his visual function and quality of life.

This case report presents various limitations. First, it is based on a single case, which limits the generalizability of the findings. Second, the study's dependence on a single follow-up visit restricts insights into long-term outcomes and potential complications. Third, the absence of patient-reported outcome measures (PROMs) prevents a comprehensive assessment of the patient's subjective experience, including satisfaction, comfort, and quality of life. Fourth, the lack of standardized fitting protocols for corneal RGP lenses in keratoconus, due to the variability in corneal tomography and eyelid geometry among patients, limits the reproducibility of the study's findings. Fifth, the study lacks a comparative analysis, which limits the understanding of the specific benefits of large-diameter corneal RGP lenses.

The results of this study suggest that large-diameter corneal RGP contact lenses are an effective management option for some keratoconus patients. However, future studies should utilize validated patient-reported outcome measures (PROMs), such as the National Eye Institute Visual Function Questionnaire-25 (NEI VFQ-25) or Ocular Surface Disease Index (OSDI), to assess the impact of these lenses on quality of life improvements. Additionally, comparative studies between small- and large-diameter corneal RGP lenses are needed to determine the optimal lens type for different keratoconus patient profiles. Furthermore, long-term follow-up studies (12 to 24 months) are crucial to evaluate the durability of management effects and identify potential long-term outcomes.

## Conclusions

This case study demonstrates the successful use of large-diameter corneal RGP contact lenses in managing a keratoconus patient. This approach accomplished excellent centration and less interaction between the lid margin and the lens edge, resulting in the best vision and patient comfort. While corneal RGP lenses of small diameter are more commonly used for keratoconus, large-diameter lenses present advantages in terms of stability and comfort. This case report underscores the potential of large-diameter corneal RGP lenses as a valuable choice for keratoconus management, especially in cases where smaller lenses fail to provide an optimal fit.
